# 748. *CoVETED Study:* Contactless Vitals for Effective Triaging in Emergency Department – A System to Reduce Cross-Infection

**DOI:** 10.1093/ofid/ofad500.809

**Published:** 2023-11-27

**Authors:** D A N T U L U R U M U R A L I D H A R VARMA, Sidharth Abrol, Mukul Rocque, Benoit Balmaekers, Navaneetha Krishnan, Kunal Gajwani, Nagaraj Poojary, Nanda Krishna Bhat, Nisarg Karanth, Sulochana Badagabettu, Reshma Pai, Prithvishree Ravindra

**Affiliations:** Department of infectious diseases, KMC Manipal, MAHE, PERAMPALLI UDUPI, Karnataka, India; Philips Innovation Campus, Bengaluru, Bengaluru, Karnataka, India; Philips Research Eindhoven, Eindhoven, Noord-Brabant, Netherlands; Philips Research Eindhoven, Eindhoven, Noord-Brabant, Netherlands; Philips Innovation Campus, Bengaluru, Bengaluru, Karnataka, India; Philips Innovation Campus, Bengaluru, Bengaluru, Karnataka, India; MAHE, Manipal, Karnataka, India; KMC Manipal, MAHE, Manipal, Karnataka, India; KMC Manipal, MAHE, Manipal, Karnataka, India; MCON Manipal, Manipal, Karnataka, India; KMC Manipal, MAHE, Manipal, Karnataka, India; KMC Manipal, MAHE, Manipal, Karnataka, India

## Abstract

**Background:**

Accuracy and speed of recording vitals are crucial for emergency department (ED) triaging. Traditionally, vital signs measurements are either skipped or recorded using non-standardized contact-based devices. ED visits sharply dropped during the COVID pandemic due to fear of cross-infection risk among patients and healthcare workers. To address this issue, we developed the "Contactless MONitoring (CMON) Spot-check Health kiosk" as a first-line screening tool to measure body temperature (T), pulse rate (PR), and respiration rate (RR) to triage patients accurately and quickly.

**Methods:**

A prospective observational study with 326 walk-in ED patients was conducted to compare the effectiveness of contactless vitals-based triaging to the current standard of care. After consent, a sitting subject's PR, RR, and T were recorded for 1 minute in a closed kiosk using non-contact cameras. For comparison, the FDA-approved contact-based PR and RR from the Philips Alice NightOne and T from an oral digital thermometer were used. Recorded data was annotated by 3 expert doctors to retrospectively triage subjects and create ground truth based on consensus. Other contact-based vitals (SpO2 and blood pressure, BP) were also included in the data annotated by the doctors. The ED nurse triaged the patients in real-time, which served as the baseline compared to the ground truth. Cohen’s Kappa (K) was used to evaluate triaging accuracy.

**Results:**

Contactless vitals (PR,RR,T) with or without patient history (Figure 1) had better triaging accuracy than the current practice of ED entrance nurse (K=0.42or 0.49 vs K=0.16). Also, patient history, when included with vitals, improves accuracy across all sets annotated, compared to vitals alone. The study also showed that CMON had good accuracy in measuring PR, RR and T (non-contact K=0.42vs contact-based K=0.43), while being acquired 20% faster.

**Figure 1:**
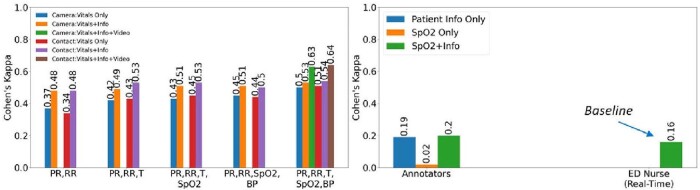
Triaging accuracy using vitals (non-contact/contact-based) with and without patient history for various vitals combinations (left), and data available to ED nurse in real-time (right)

**Conclusion:**

Contactless monitoring can improve patient triaging by providing more frequent and accurate vital sign monitoring, allowing for better-informed decisions about patient care. When combined with patient information and integration with Electronic Medical Record systems, it can efficiently manage patient footfall and reduce cross-infection risk.

**Disclosures:**

**Sidharth Abrol, PhD**, Philips: Employed by Philips|Philips: Stocks/Bonds **Mukul Rocque, MS**, Philips Electronics Nederland B.V.: Multiple patents in the field of patient monitoring|Philips Electronics Nederland B.V.: Employee|Philips Electronics Nederland B.V.: Stocks/Bonds **Benoit Balmaekers, MSc**, Philips Electronics Nederland B.V.: Patent in the field of respiration measurement|Philips Electronics Nederland B.V.: Employee|Philips Electronics Nederland B.V.: Stocks/Bonds **Navaneetha Krishnan, MS**, Philips: Employee **Kunal Gajwani, MS**, PHILIPS: EMPLOYEE

